# Sequential Patterns of Multimorbidity in a Nationally Representative Cohort Study of Aging

**DOI:** 10.1093/geroni/igaa057.718

**Published:** 2020-12-16

**Authors:** Nicholas Schiltz, Yicheng Hu, Megan Foradori, Jessica Cooke Bailey

**Affiliations:** 1 Case Western Reserve University, Cleveland, Ohio, United States; 2 Case Western Reserve University School of Medicine, Cleveland, Ohio, United States

## Abstract

Multimorbidity – the presence of two or more chronic health conditions – is common among older adults. Despite this, relatively little is known about the epidemiology of specific sequences of disease onset that occurs in mid-life and older adults over time. This may be attributed to the sheer number of possible permutations, which is difficult to handle with traditional methods. This is a retrospective cohort study using the Health & Retirement Study (HRS), a nationally-representative panel survey of aging. The study population included all adults age 50 and older that had no reported chronic disease at baseline (n=5567). We use a data mining algorithm, Sequential Pattern Discovery using Equivalence classes (SPADE), to identify all possible sequences of eight self-reported age-related chronic diseases: hypertension, arthritis, diabetes, cancer, stroke, heart disease, chronic lung disease, and psychiatric disorders. There were 67 unique sequences of disease identified that occurred in at least 1% of the study population. The most common two event sequence was Arthritis=>Hypertension (15.5% of all subjects), and the second most common was Hypertension=>Arthritis (9.6%). The most common three-way sequence was Arthritis=>Hypertension=>Heart Disease (1.8%). Arthritis=>Stroke occurred in 1.5% of subjects and was associated with the highest mortality rate (71.3% of subjects died). Sequential pattern mining allows for the discovery of longitudinal patterns of disease that frequently occur in older adults and advancements in our understanding of the epidemiology of multimorbidity. Future applications may include predicting a given patient's disease trajectory based on their life course and disease history.

